# Promising therapeutics of enterovirus 71 infection: sunshine behind cloud

**DOI:** 10.3389/fphar.2025.1652159

**Published:** 2025-09-16

**Authors:** Sheng-Yu You, Shun-Hua Chen, Shih-Min Wang

**Affiliations:** ^1^ Department of Microbiology and Immunology, College of Medicine, National Cheng Kung University, Tainan, Taiwan; ^2^ Center of Infectious Disease and Signaling Research, National Cheng Kung University, Tainan, Taiwan; ^3^ Institute of Basic Medical Sciences, College of Medicine, National Cheng Kung University, Tainan, Taiwan; ^4^ Center for Infection Control, National Cheng Kung University Hospital, College of Medicine, National Cheng Kung University, Tainan, Taiwan; ^5^ School of Medicine, College of Medicine, National Cheng Kung University, Tainan, Taiwan

**Keywords:** enterovirus 71, brainstem encephalitis, pulmonary edema, antiviral, entrovirus

## Abstract

Enterovirus 71 (EV71) infection gave a hard hit on young children because of fatal complication, brainstem encephalitis with pulmonary edema. The occurrence of severe EV71 infections highlight the urgent need for the development and repurposing of novel antivirals for medical use. Drugs target specific steps in the cycle of viral replication, and the modification of existing factors in EV71 immunopathogenesis deciphers the current approaches for developing antivirals. In addition to identifying chemical compounds, we highlight active constituents and explore underlying mechanisms of action of antimicrobial peptides and natural products that may be active against EV71 and provide pharmacological benefits.

## 1 Introduction

International travel, economics, cultural interactions, and climate change have moved much of the world into a becoming a global village. Viruses, which are potential silent reservoirs for emerging and re-emerging diseases that may lead to global outbreaks, have political, economic, and social impacts. For example, the pandemic of coronavirus disease 2019 (COVID-19) caused by the novel severe acute respiratory syndrome coronavirus 2 (SARS-CoV-2), presented an unprecedented challenge to managing a global contagion (Weston et al., 2020). Enteroviruses infections can cause systemic dissemination and central nervous system (CNS) diseases, become emerging or re-emerging epidemics and pose a heavy societal burden. The initial outbreak of enterovirus 71 (EV71) infections in Taiwan was in 1998, with about 1.5 million infected children. The prominent complication of CNS in EV71 infection is brainstem encephalitis (BE) (Wang, 2016). The EV71 BE is reported to be the most serious neurological manifestation of enterovirus infections, may progress to autonomic nervous system (ANS) dysregulation, neurogenic pulmonary edema (NPE) and cardiopulmonary failure (Wang, 2016). With regard to the previous pioneer and novel findings on EV71 BE and severe complications, further investigation for the potential effectiveness and efficacy of treatments of EV71 infections is life-critical.

## 2 Clinical manifestations

Enterovirus 71 (EV71) belongs to the genus Enterovirus which causes multiple mild-to-severe clinical illnesses, including herpangina, hand, foot, and mouth disease, meningitis, brainstem encephalitis, autonomous nervous system (ANS) dysregulation, and pulmonary edema in young children (Wang, 2016). EV71 consists of a non-enveloped, single-stranded, positive-sense RNA within the family of *Piconaviridae*. EV71 is divided into four genotypes, A, B, C and D. The capsid contains four coat proteins, including VP1, VP2, VP3, and VP4. Variations within capsid proteins VP1 to VP3 are responsible for the antigenic diversity, and neutralization sites are most clustered on VP1 (Liu and Long, 2025). EV71 is similar to many emerging and re-emerging viruses with specific neuro-invasive targets, such as the brainstem. Since 1997, EV71 has emerged as a serious childhood infection in the Asia-Pacific region (Modlin, 2007). Based on clinical, laboratory, radiological, and pathological evidence, EV71 was the cardinal pathogen of brainstem encephalitis during the first outbreak in 1998, in Taiwan (Wang, 2016).

## 3 Immunopathogenesis

The immunopathogenesis of EV71 has been studied in terms of its width and depth for the diagnosis and clinical treatment during the past 25 years (Wang et al., 2003). Humoral mediators, including cytokines, play key roles in the pathophysiology of critical diseases caused by microbiological pathogens. Cytokine storm surges occur and persist in the condition known as hypercytokinemia, resulting in progression to multiple organ failure. Previous studies have clearly demonstrated that cytokines and chemokines play important roles in the pathogenesis of EV71 severe complications. Both CNS (IL-1β, IL-6, IFN-γ, IP-10, MIG) and systemic inflammatory responses (IL-6, IL-8, IL-10, IL-13, IP-10, MCP-1, MIG) to infection play leading, but distinctly different, roles in the pathogenesis of EV71 pulmonary edema (Wang et al., 2003; Wang et al., 2007). Cellular immunity also contributes to the pathogenesis of complications arising during EV71 infection. EV71-infected patients with pulmonary edema have fewer circulating CD4^+^ T cells, CD8^+^ T cells, and natural killer cells (Wang et al., 2003). Furthermore, CD19^+^ and CD20^+^ B cell expression frequency and absolute cell number did not change significantly during EV71 infection according to disease severity (Wang, unpublished data).

## 4 Therapeutics options, past and current perspectives

Optional therapeutic measures for viral diseases include the use of antiviral agents, neutralizing antibodies, and vaccines to reduce disease severity and mortality. Although EV71 vaccination alleviated the severity, incidence and altered epidemiological trends. Whereas, a limitation of EV71 vaccines is lack of geographic scope, to generalize the efficacy and effectiveness of vaccines against EV71 to every epidemical regions or countries (Swain et al., 2022).

Previous clinical experiences with EV71 infection treatment are discussed first. Intravenous immunoglobulin (IVIG) has been therapeutically used against EVs in the clinical settings of neonates and children, depending on the serotypes of infection (Rotbart, 2000). IVIG administration neutralizes specific EV, alters immunoregulatory molecules, and reduces inflammatory mediators to attenuate the disease course in EV71-infected patients with ANS dysregulation. IVIG consists of antigen-specific IgG antibodies that can move to a population with reduced sialic acid levels, which are capable of mediating antigen clearance and a protective inflammatory response through the engagement of subclass-specific FcγRs on effector cells (Kaneko et al., 2006). Milrinone is a subtype III cyclic nucleotide phosphodiesterase (PDE) inhibitor that increases intracellular cyclic adenosine monophosphate (cAMP) levels. Milrinone treatment has both inotropic and vasodilatory effects and is associated with decreased mortality through increased regulatory T cell populations, cAMP expression, and modulation of cytokine levels in EV71-infected patients (Wang, 2016). Furthermore, treatment with EV71-specific antibodies before or after infection considerably reduced the disease severity, mortality, and tissue viral load in B cell-deficient mice. Above treatments indicated that both lymphocyte and antibody responses protected mice from EV71 infection (Lin et al., 2009). Recently, Yang et al. discovered that the recruitment of CCL3-mediated neutrophils to the brain is critical in the fatal immunopathology of EV71 infection, suggesting that CCL3 is a potential therapeutic target that downregulates the detrimental roles of neutrophils during EV71 infection (Yang et al., 2024).

Clinically, no antiviral agents have been approved by the Food and Drug Administration (FDA) for the treatment of EVs. Sustained development of drugs for the treatment of picornaviral infections is essential. Research may focus mainly on the viral capsid, viral proteases, or replication inhibitors targeting other non-structural enteroviral proteins. Entry is the first step in viral infection and involves viral attachment, endocytosis, and uncoating. Binding of the virus to its receptor is critical for tropism of the virus. The design of novel antiviral agents that target specific steps in the viral replication cycle and modification of existing antiviral compounds are current approaches for developing antiviral drugs (Abzug et al., 2003).

Pleconaril, a capsid function inhibitor, was the first compound to exhibit broad-spectrum anti-EV activity (Abzug et al., 2003). However, pleconaril treatment failed to show consistent efficacy in patients with EV meningitis or meningoencephalitis in a randomized double-blind placebo-controlled trial (Norder et al., 2011). Ribavirin is a nucleoside analog with broad-spectrum antiviral activity against various viruses. Ribavirin effectively reduced viral production *in vitro* and also reduced viral loads in tissues, as well as mortality, morbidity, and subsequent paralysis sequelae in an ICR mouse model (Li et al., 2008). Minocycline has neuroprotective effects through anti-inflammatory, anti-apoptotic, and immunomodulatory properties (Bastos et al., 2013). It possesses high lipophilicity and is easily transferred through the blood-brain barrier to accumulate in both cerebrospinal fluid and neurons. We clearly demonstrated that clinical scores, mortality rates, and viral titers in various brain tissues decreased after minocycline treatment in an MP4-infected animal model (Liao et al., 2019). Minocycline inhibited IL-6 and granulocyte colony-stimulating factor (G-CSF) in plasma and TNF in the cerebellum. Minocycline has characteristics and properties that function both as an antiviral and anti-inflammatory agent in EV71 infection (Liao et al., 2019). Antimicrobial peptides (Tripodi et al.) are important effectors of the innate immune system and are critical defense mechanisms against various pathogenic infections in humans (Huan et al., 2020). Cathelicidin peptide analogs were effective against EV71 infection, but could not inhibit echovirus or coxsackievirus, suggesting that these peptides are highly specific. LL-18 and FF-18, which belong to the cathelicidin family, are effective against EV71 infections *in vitro* and *in vivo*. These peptides bind directly to the viral particle surface and prevent the virus from entering the host cell, thereby blocking viral genome release (Fan et al., 2024). Scorpion venom antimicrobial peptide BmKn2, isolated from the scorpion *M. martensii*, and its derivative BmKn2-T5, have a typical amphiphilic α-helical structure and exhibit significant inhibitory activity against EV71 during early stages, especially in attachment and entry (Xia et al., 2024).

Numerous herbal compounds and their bioactive metabolites have become a significant focus of research because of their efficacy and cost-effectiveness (Bachar et al., 2021). The EV71 2A protease (2Apro), a cysteine protease produced by the virus, is essential for viral replication (Lin et al., 2019). It is commonly isolated from the roots and rhizomes of *Podophyllum* spp. Etoposide inhibited EV71 replication in a concentration-dependent manner in various cell lines with minimal cytotoxicity. Etoposide inhibits the activity of EV71 2A protease by binding mainly to two residues: Y89 and P107 (Liang et al., 2025). Magnolol, which is extracted from *Magnolia officinalis*, is known for its antitumor, anti-inflammatory, and antioxidant effects (Lee et al., 2011). Magnolol demonstrated significant dose-dependent inhibition of EV71 replication *in vitro*. It attenuated viral load in brain and limb tissues and mitigated tissue inflammation. It upregulated the overall expression of nuclear factor erythroid 2 - related factor 2 (Nrf2) and facilitated its nuclear translocation, resulting in increased expression of various ferroptosis inhibitory genes (Zhao et al., 2024). *Grifola frondosa* is an important medicinal and edible fungus rich in flavonoids, fatty acids, and other active substances (Tripodi et al., 2022). L-tryptophan has been used for the separation and purification of *Grifola frondosa*. Xiong et al. found that L-tryptophan has high anti-EV71 activity and reduced EV71-induced apoptosis. It significantly inhibited the replication process after viral adsorption by binding to the VP1 capsid protein, preventing the virus from attaching to and entering cells (Xiong et al., 2024). The information on various aspects of the promising compounds, including the functional pathways and mechanisms were summarized in Table 1. There is no clinical approved natural products currently available for the prevention and treatment of the EV71 infections. One of the technical block is to develop an amendable assay for high-throughput screening (Wang et al., 2015).

**TABLE 1:** Selected current agents with therapeutic potential against enterovirus 71 infections.

Agent	Advantages	Pathways/Mechanisms	Models	References
Minocycline	1. Antiviral 2. Anti-inflammatory	1. Decreased viral RNA, viral protein, replication and tissue viral loads 2. Decreased G-CSF, IL-12p40, IL-1β, IL-6, IL-8 and TNF expression 3. Decreased mortality and disease severity	1. *In vitro*: U-87 MG, RD, THP-1 cell 2. *In vivo*: ICR mice	Liao et al. (2019)
Cathelicidin peptide analogues	1. Antiviral	1. LL-18 and FF-18 inhibit viral attachment and internalization 2. Antiviral effect of LL-18 is 20th amino acid residue 3. Low propensity of LL-18 to induce virus resistance	1. *In vitro*: 293T and RD cell 2. *In vivo*: ICR mice	Fan et al. (2024)
BmKn2 and BmKn2-T5	1. Antiviral	1. Decreased viral RNA, viral protein, viral attachment and viral loads	1. *In vitro*: RD cell	Xia et al. (2024)
Etoposide	1. Antiviral	1. Inhibited activity of EV71 2A protease	1. *In vitro*: RD, HEK-293T, Vero cell	Liang et al. (2025)
Magnolol	1. Antiviral 2. Anti-oxidative 3. Improves survival rate	1. Decreased viral RNA, viral protein, and tissue viral loads 2. Upregulated Nrf2 and facilitated nucleus translocation 3. Simulated Nrf2-SLC7A11-GSH axis to inhibite ferroptosis	1. *In vitro*: RD, HeLa cell 2. *In vivo*: ICR mice	Zhao et al. (2024)
Active components of *Grifola frondosa* (L-tryptophan)	1. Antiviral	1. Reduced EV71-induced apoptosis 2. Inhibited replication process 3. Prevent virus from attaching cells	1. *In vitro*: Vero cell	Xiong et al. (2024)

## 5 Conclusion

Drug repurposing is a way to discover at existing drugs function to extent if they could be used to treat other diseases. These agents may represent potential roles of new therapeutics for controlling or preventing EV71 complications. Further, evaluate the therapeutics for EV71 infection is undergoing in animal models. An extensive future investigation is also required with respect to the mechanisms of action at systemic, cellular, and molecular level to extend up to broad-spectrum clinical trials. The resurgence of EV71, particularly its association with complications, including brainstem encephalitis, autonomous nervous system (ANS) dysregulation, and pulmonary edema in patients, underscores the urgent need for effective antiviral strategies against virulent strains that may emerge, in addition to vaccines.
